# Monthly Summary

**Published:** 1898-01

**Authors:** 


### Monthly Summary.

OFFICE DROWSINESS.—"Some men are quite martyrs to office drowsiness," a physician remarked the other day. "Any monotonous sound near them, the hum of traffic outside, or even the scratching of a clerk's pen is sufficient to induce a feeling of sleepiness which it is almost impossible to resist."

The worst of it is that this symptom is so seldom regarded as anything serious, though I have known it to be the beginning of critical mental trouble. For more often, however, it is merely the effect of constitutional eccentricity though in either case a few simple remedies might be tried with advantage.

For instance, I always advise the old indigestion cure—a glass of hot water—when the feeling comes on. To keep the eyes tightly closed for three or four minutes and then bathe them in very warm water often gives relief at once. And another good idea is to lower the head for a few seconds to a level with the knees. Above all, one should never give in to the feeling of drowsiness by taking a short nap in the hope of waking up brighter after it.

At the same time the condition of the office might be looked into. The slightest defect in ventilation will often cause one man to be affected by office drowsiness, even though other people in the same room feel nothing of it whatever.—*Tid-Bits.*

---

THE TIME was when many of the leading dentists of this country were, on what they considered to be unassailable scientific grounds, opposed to the use of amalgam, and opposition to it was the leading thought of most of the early societies. Every society that actively opposed the use of amalgam has ceased to be. Men of enlightened minds insist on doing their own thinking, free and untrammeled, and in a mutual society they insist on, without wide limits, the right to express, without being thereby made objects of ridicule or offensive remarks, their own opinions. Any society, which either by rule or license abridges this right does so at its peril.—W. H. Trueman, in *Review.*

---

MOST of the dentists, particularly the young men, are using the engine entirely too much. I do not wish to have the impression made that I do not use the engine at all, because I do, and I would not like to be without it. I use a sharp excavator with a handle about six inches long. I clean out my cavities with it. I use the engine in the preparation of some cavities. If I want to cut out a fissure of some kind, or to cut tissue, I use the engine for that, with fissure drill or bur.—Dr. Templeton, in *Review.*

---

DEAFNESS.—It has taken the medical world many years to discover that loss of hearing is almost invariably caused by some disease of the throat or nose or oral cavity. But very recent researches in these fields have demonstrated this fact beyond question, and it is now admitted by the most advanced medical men that, aside from the rupture of the ear drum, there is scarcely a symptom of defective hearing that is not traceable to the condition oi these cavities.—*Practitioner.*

